# An Investigation into a Gear-Based Knee Joint Designed for Lower Limb Prosthesis

**DOI:** 10.1155/2017/7595642

**Published:** 2017-05-05

**Authors:** M. S. H. Bhuiyan, I. A. Choudhury, M. Dahari, Y. Nukman, S. Z. Dawal

**Affiliations:** ^1^Manufacturing System Integration, Department of Mechanical Engineering, Faculty of Engineering, University of Malaya, 50603 Kuala Lumpur, Malaysia; ^2^Department of Electrical Engineering, Faculty of Engineering, University of Malaya, 50603 Kuala Lumpur, Malaysia

## Abstract

A gear-based knee joint is designed to improve the performance of mechanical-type above-knee prostheses. The gear set with the help of some bracing, and bracket arrangement, is used to enable the prosthesis to follow the residual limb movement. The motion analysis and finite-element analysis (FEA) of knee joint components are carried out to assess the feasibility of the design. The maximum stress of 29.74 MPa and maximum strain of 2.393e−004 are obtained in the gear, whereas the maximum displacement of 7.975 mm occurred in the stopper of the knee arrangement. The factor of safety of 3.5 obtained from the FE analysis indicated no possibility of design failure. The results obtained from the FE analysis are then compared with the real data obtained from the literature for a similar subject. The pattern of motion analysis results has shown a great resemblance with the gait cycle of a healthy biological limb.

## 1. Introduction

For an above-knee amputee, the prosthetic knee joint is a vital element, which plays a complex role in providing stability to its users in the absence of knee extensors [1]. The mechanical knee joints available for prostheses are cheap and robust; however, they are unable to follow the real-time movement of the residual limb. The advanced microprocessor-controlled knee joints have achieved a very good command in recreating desired movement in the prosthesis by compromising with the price [2]. The newly designed knee joint will enhance the controllability of the mechanical-type prosthesis within an affordable price. Unlike the typical mechanical knee joints, the proposed design will make the shank follow the residual limb movements without having any external power supply. There is a set of gears integrated to the thigh and shank, a bracket holding the gear set, and a waist belt helping the bracket to restrict its movement. The entire arrangement collectively will allow the knee to move following the residual limb movement.

The typical mechanical-type lower limb prostheses are unable to follow the residual limb movement properly, whereas the electrical and hybrid-type prostheses require external power supply to operate the prosthesis. The power is usually supplied by some batteries, which require charging after a certain period of time and are heavy to carry for the amputee. Besides, the mechanically controlled prostheses are robust and cheap and are affordable to a large group of amputees, whereas the advanced-type microprocessor-controlled prostheses are expensive and beyond reach for the majority proportion of amputees. The proposed design is unique and remarkably different from other types of existing prostheses due to its ability of following the residual limb movement without having any external power supply.

A thorough analysis on gait kinematics, kinetics, and energetics could help evaluate the performance of the knee joint in an above-knee prosthesis. The joint angular movement is assessed by kinematics analysis whereas the ground reaction forces and internal joint moment are observed by kinetics analysis, and the joint power is evaluated by energetics analysis [3]. The gait mechanics and the compensatory mechanisms adopted by the amputee walking with an above-knee (AK) prosthesis are determined by the joint kinetics [4]. The finite-element analysis of knee joint components would provide an idea on knee joint kinematics and thus enable the designers to predict the component failure by analyzing the simulation results. Using FEA, Zach et al. [5] have analyzed the plastic stability and von Mises stress of the material and the contact pressure distribution on the components' surfaces during knee-bending in 28.41°. The variations of the von Mises stress distributions in bipolar hemi-knee prosthesis and unipolar prosthesis were observed by Lian et al. [6] when the prosthesis was moved at a different gait cycle under static upright posture. Maximum stress values were also determined based on the dynamic analysis of the unipolar and bipolar joint prostheses.

This study investigates kinematics, kinetics, and structural changes of prosthetic knee joint components by performing motion analysis and finite-element analysis on the SOLIDWORKS platform. As the prosthesis is designed for level-ground walking, the motion analysis is carried out to ascertain the changes in kinematics and kinetics of the proposed gear-based knee joint during level-ground walking only. The finite-element analysis of different knee joint components is conducted to observe the material structural/physical changes during the gait cycle. A comparative study between the simulation results and the real data captured from a healthy subject is also carried out to validate the design.

## 2. Design of Knee Joint

The proposed knee joint design has key features that are dissimilar to other currently available knee joints for prosthetic legs. The typical mechanical knee joints are hinge-type joints, whereas the newly designed knee joint is comprised of two spur gears, two bushing pins, and two bracing plates. The gears are mounted on the bushing pins without having locked to the pins. However, these are not free to rotate about the bushing pins because of their connection with the thigh and shank rods. One of the two gears is connected with the lower ends of the thigh rod, and another is integrated to the upper end of the shank rod. They are kept engaged using a set of bearing plates. Each bushing pin, on the other hand, is mounted on two ball bearings from two ends, which are themselves mounted on the bearing plates. The gears set with the bearing plates are hold together with a U-shaped bracing. The lower end of a bracket is bolted with the U-shaped bracing whereas the upper end of the bracket is attached to a waist belt. The U-shaped bracing is used to assist the bracket holding the gear set firmly during movements. A plano-concave-shaped piece of material is used at the joint in between the bracket and waist belt to keep the bracket firm and not moving during flexion and extension of the knee. The mating gears with the help of the bearing plates, brace, bracket, and waist belt will make the knee move following the residual limb movement and thus enable the prosthesis to move according to the user's intensions. A set of stopper is screwed to the faces of each gear. These are incorporated into the design to prevent further movement of the gears when the user is at the stance phase and thus help the amputee to stay upright.

**Figure 1:**
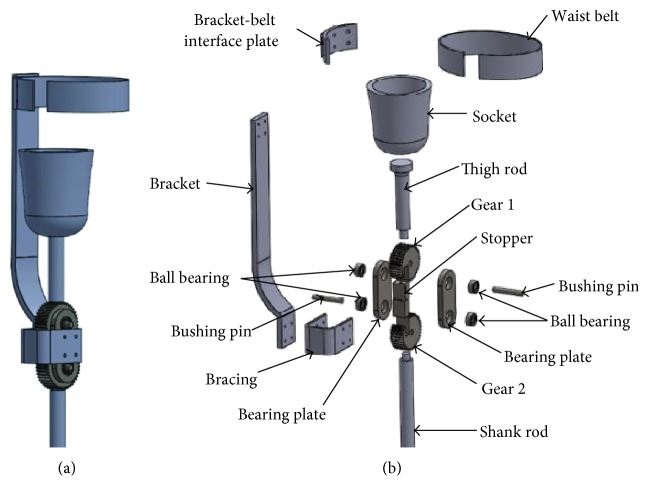
(a) Isometric view and (b) exploded view of the knee joint components.

The additional bracket-bracing arrangement attached to the waist of the amputee and the weight of the prosthesis together would help the prosthesis to balance during standing and walking phases. The different components of the gear-based knee joints, including the guiding arrangement, are shown in Figure 1.

The knee joint assembly is designed to reproduce a similar movement like that of a healthy biological limb in the above knee prosthesis. The gear set with the help of the bracket and bracing arrangement would make the shank follow the residual limb movement rather than just allow it to bend. This phenomenon will make the knee joint behave like an active knee joint. Though the gear-based knee joint is technically a passive joint, due to its behavior like an active joint, it is named as a quasi-active knee joint.

## 3. Motion Analysis of Knee Joint

Motion analysis is performed to determine how an assembly and its components move physically under an applied load. Structural analysis is carried out to see the possible changes in the design components during operation. The structural analysis is usually performed based on the force measurement and the motion study of the assembly. In the motion study, both the kinematic and the kinetics analyses are carried out.

### 3.1. Kinematic Analysis

Kinematic analysis is carried out to study the motion of objects when the causes of the changes are put out of consideration. To determine the range of motion of the assembly, displacement, velocities, and accelerations of the components is the key result of interest. For knee joint kinematic analysis, the angular displacement and angular velocity under force and the torque are observed, which are then compared with the practical test results to see the deflections. A transfemoral amputee with a mass of 69 kg and height of 166.5 cm is taken as a subject. At the stance phase of the gait cycle, the applied load on the knee gears is entirely from the body weight of the amputee, and the value of applied torque will be zero. However, at the swing phase, the applied load will be the *Y*-component of the amputee body weight for the accrued angle of rotation by the knee, and the value of the applied torque will be the torque due to the *X*-component of amputee body weight for that particular angle of rotation.

The variables used in the simulation are based on the features of the subject, which are tabulated in Table 1.

The weight of the subject was 676.9 N.

Applied load = weight of the subject + weight of the thigh rod and socket arrangement = resultant weight = *W*_r_ = 679.9 + 1.5∗9.81 = 691.6 N

The proportion of the subject weight is shared equally by both legs while standing, which is altered with the changes of foot contact with the ground during the swing phase. For the sake of simplifying the calculation, it was presumed that the weight was shared equally by the two legs; therefore, the weight should be divided by two. However, in the event of balance losing, the body weight becomes 1x to 4x that of the real body weight. Therefore, the total weight was considered as the applied load.

**Figure 2:**
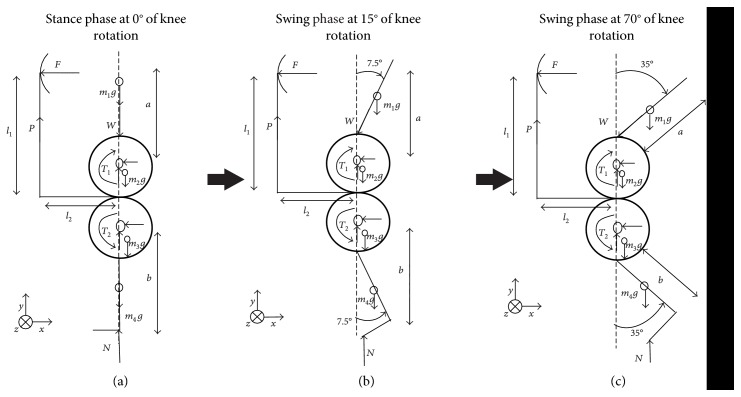
Free-body diagram of a prosthetic knee joint at different phases of the gait cycle.

Figure 2 shows the free-body diagram of a prosthetic knee joint at different phases of the gait cycle.

At the stance phase of the gait cycle, there is no rotation at the knee joint. The following equilibrium equations can be obtained from the free-body diagram of Figure 2.

For the stance phase at 0° of knee rotation, the equilibrium equations are as follows: force equilibrium (∑*F*_*X*_ = 0; ∑*F*_*Y*_ = 0),

(1) $$\begin{array}{c} W+\left(m_{1}+m_{2}+m_{3}+m_{4}\right)\;g-P-N=0, \end{array}$$

moment equilibrium (∑*M*_*X*_ = 0; ∑*M*_*Y*_ = 0),

(2) $$\begin{array}{c} Fl_{1}-T_{1}+T_{2}-Pl_{2}=0,\\ T_{1}=0,\\ T_{2}=0,\\ P=0, \end{array}$$

where *W* = body weight of subject, *m*_1_ = weight of thigh rod, *m*_2_ = weight of gear 1, *m*_3_ = weight of gear 2, *m*_4_ = weight of shank rod, *g* = gravity, *P* = pulling force applied by the guiding arrangement, *N* = ground reaction force, *F* = resistance force by the guiding arrangement, *F*_r_ = resultant force, *l*_1_ = distance from waist belt to knee joint, *l*_2_ = distance from bearing plate to bracket arm, *T*_1_ = torque at gear 1, *T*_2_ = torque at gear 2, and T_r_ = resultant torque.

During the swing phase of the gait cycle, the knee of the subject is found to rotate from 15° to 70°, which has to be imitated by the prosthetic knee joint.

For the swing phase at 15° of knee rotation, the equilibrium equations are as follows: force equilibrium (∑*F*_*X*_ = 0; ∑*F*_*Y*_ = 0),

(3) $$\begin{array}{c} W\;\cos\;15+\left(m_{1}+m_{2}\right)g-N\;\cos\;15+\left(m_{3}+m_{4}\right)\;g-P=F_{r}, \end{array}$$

moment equilibrium (∑*M*_*X*_ = 0; ∑*M*_*Y*_ = 0),

(4) $$\begin{array}{c} Fl_{1}-T_{1}+T_{2}-Pl_{2}=T_{r},\\ T_{1}=W\;\sin\;15\;*\;a,\\ T_{2}=N\;\cos\;15\;*\;b. \end{array}$$

For the swing phase at 70° of knee rotation, the equilibrium equations are as follows: force equilibrium (∑*F*_*X*_ = 0; ∑*F*_*Y*_ = 0),

(5) $$\begin{array}{c} W\;\cos\;70+\left(m_{1}+m_{2}\right)\;g-N\;\cos\;70+\left(m_{3}+m_{4}\right)\;g-P=F_{r}, \end{array}$$

moment equilibrium (∑*M*_*X*_ = 0; ∑*M*_*Y*_ = 0),

(6) $$\begin{array}{c} Fl_{1}-T_{1}+T_{2}-Pl_{2}=T_{r,}\\ T_{1}=W\;\sin\;70\;*\;a,\\ T_{2}=N\;\cos\;70\;*\;b,\\ N=0. \end{array}$$

Therefore, the *Y*-component of the subject's weight at 15° of rotation can be derived as follows.

Torque arm length for the femur at 15° and 70° of rotation was *l*_1_ = 515 mm; torque arm length for the tibia at 15° and 70° of rotation was *l*_2_ = 410 mm.

During the swing phase of the gait cycle, the subject's knee is found to rotate from 15° to 70°, which is to be imitated by the prosthetic knee joint. The angle of rotation is the resultant angle of both the gears. Therefore, to generate 15° of rotation, each gear has to rotate 7.5°; and to create 70° of rotation, each gear has to rotate 35°.

Therefore, the value of the *Y*-component of the subject weight at 15° of rotation angle can be derived as follows.

At the stance phase of the gait cycle, 0° of rotation of the knee joint, the applied load on both the gears of the knee joint = *Y*-component of the resultant weight = applied load = 691.6 N.

The *X*-component of the subject body weight = 0 N.

Therefore, the applied torque on both the gears of the knee joint = 0 Nm.

At the swing phase of the gait cycle, 15° of rotation of knee joint, the applied load on gear 1 of the knee joint = *Y*-component of the resultant weight at 7.5° of rotation:

(7) $$\begin{array}{c} F_{Y15deg}=F\;\cos\;7.5=691.6\;*\;\cos\;7.5=685.7\;N. \end{array}$$

The applied load on gear 2 of the knee joint = *Y*-component of the subject body weight at 15° of rotation:

(8) $$\begin{array}{c} F_{Y15deg}=F\;\cos\;7.5=691.6\;*\;\cos\;7.5=685.7\;N. \end{array}$$

The *X*-component of the subject's body weight at 15° of rotation is as follows:

(9) $$\begin{array}{c} F_{X15deg}=F\;\sin\;7.5=691.6\;*\;\sin\;7.5=90.3\;N. \end{array}$$

Therefore, the applied torque on gear 1 of the knee joint at 15° of rotation is as follows:

(10) $$\begin{array}{c} \tau_{X15deg}=F_{X15deg}\;*\;l_{1}=90.3\;*\;0.515=46.5\;Nm. \end{array}$$

The applied torque on gear 2 of the knee joint at 15° of rotation is as follows:

(11) $$\begin{array}{c} \tau_{X15deg}=F_{X15deg}\;*\;l_{2}=90.3\;*\;0.410=37.0\;Nm. \end{array}$$

At the swing phase of the gait cycle, 70° of rotation of knee joint, the applied load on gear 1 of the knee joint = *Y*-component of the subject body weight at 35° of rotation.

(12) $$\begin{array}{c} F_{Y70deg}=F\;\cos\;35=691.6\;*\;\cos\;35=566.5\;N. \end{array}$$

The applied load on gear 2 of the knee joint = *Y*-component of the subject body weight at 70° of rotation:

(13) $$\begin{array}{c} F_{Y70deg}=F\;\cos\;35=691.6\;*\cos\;35=566.5\;N. \end{array}$$

The *X*-component of the subject body weight at 70° of rotation is as follows:

(14) $$\begin{array}{c} F_{X70deg}=F\;\sin\;35=691.6\;*\;\sin\;35=396.7\;N. \end{array}$$

Therefore, the applied torque on gear 1 of the knee joint at 70° of rotation is as follows:

(15) $$\begin{array}{c} \tau_{X70deg}=F_{X70deg}\;*\;l_{1}=396.7\;*\;0.515=204.3\;Nm. \end{array}$$

The applied torque on gear 2 of the knee joint at 70° of rotation is as follows:

(16) $$\begin{array}{c} \tau_{X70deg}=F_{X70deg}\;*\;l_{2}=396.7\;*\;0.410=162.6\;Nm. \end{array}$$

The boundary conditions used in the simulation of the prosthetic knee joint are shown in Table 2.

**Figure 3:**
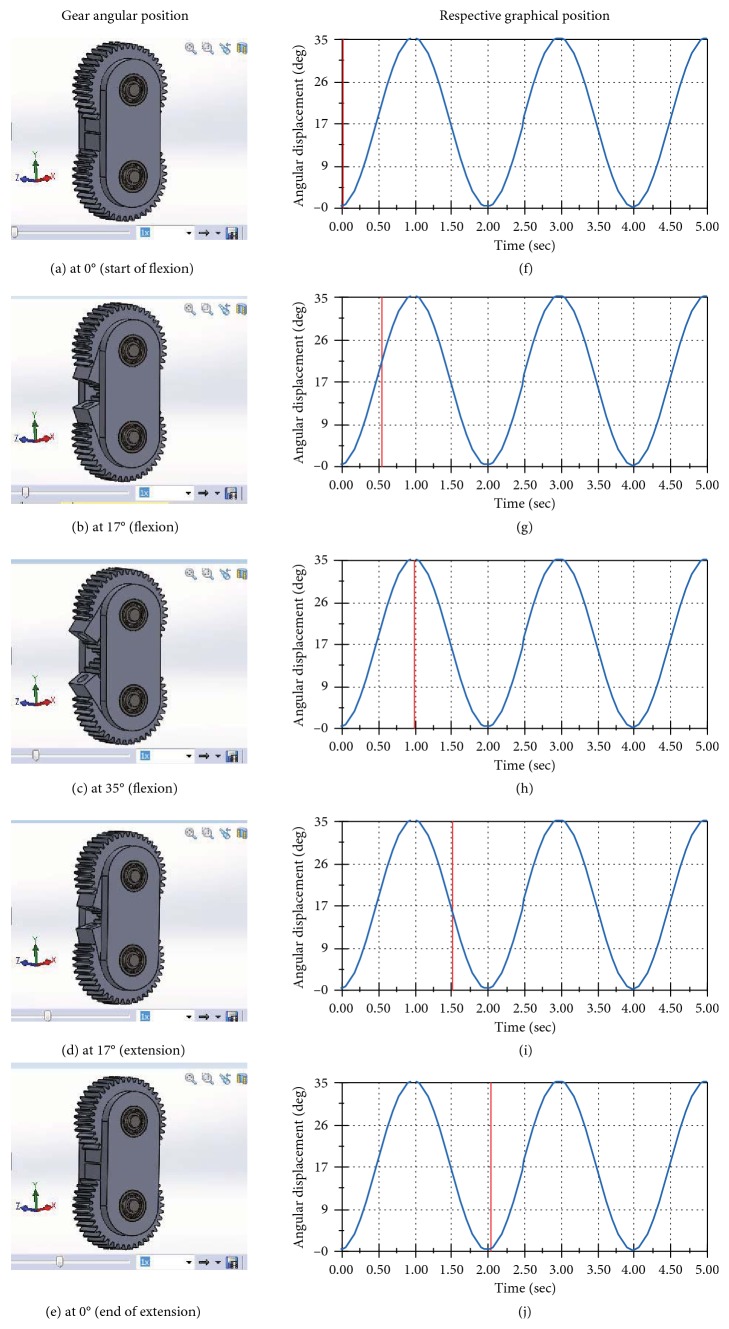
Angular position of gear at different phases of the gait cycle.

The angular displacements of the gears at different phases of the gait cycle are shown in Figure 3.

From Figure 3, the angular position of the gear at the stance phase is 0°, which gradually increases to 70° during the swing phase. Figure 3(a) shows the angular position of the gears of the knee joint at the stance phase, whereas Figures 3(b)–3(e) show the different angular positions of gears in degrees throughout the swing phase. The corresponding angular displacements are represented in the graph of Figures 3(g)–3(j).

### 3.2. Kinetics Analysis

The forces involved with the movement of the components are evaluated by the kinetics analysis. The joint moment and joint power are the important factors to be investigated in the kinetics analysis of the knee joint.

## 4. Gait Analysis

The gait cycle of a healthy lower limb was recorded prior to modeling and simulating a prosthesis design. The natural gait analysis was performed to obtain spatial, temporal, kinematics, and kinetics information of lower limb locomotion required for designing a prosthesis. The design was optimized based on these gait analysis data before fabrication. Those data were also used as reference during the performance test of the prosthesis.

### 4.1. Experimental Setup

**Figure 4:**
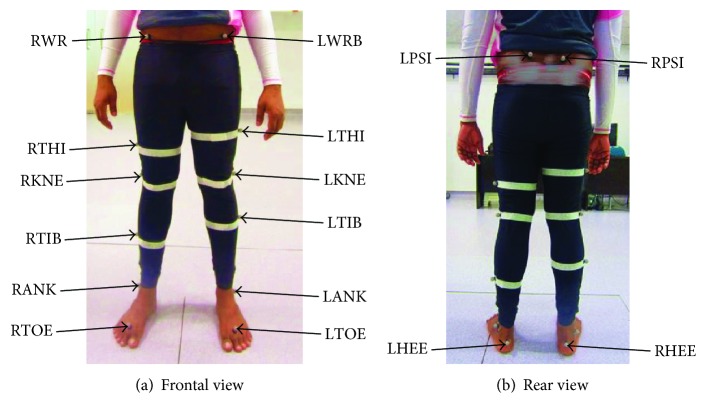
Marker positions on the lower limb of a healthy individual in gait analysis.

The results obtained from the modeling and simulations have been validated with the help of gait cycle data captured from the healthy subject. The gait cycle data from the healthy subject was captured using an arrangement of five AMEX cameras and a VICON system with the help of sixteen reflective markers placed at different locations of the joints and segments of the lower limb. The gait analysis required the subject to be equipped with reflective markers and produce movements within a defined area, which is encompassed by an arrangement of some near infrared (NIR) cameras and a data recording system. Sixteen reflective markers were placed at different locations of the joints and segments of the lower limb. A set of markers were placed at the left and right feet (LTOE and RTOE).  Figure 4 shows the placement of the markers. The exact position of the marker was at the second metatarsal head, on the midfoot side of the equinus break between the forefoot and midfoot. Then, another set of markers were placed at the calcaneus of each heel (LHEE and RHEE) at the same height above the plantar surface. At the ankle joint, markers were placed on the lateral malleolus (LANK and RANK) along an imaginary line that passes through the transmalleolar axis. The tibial markers (LTIB and RTIB) were placed over the lower 1/3 of the shank to determine the alignment of the ankle flexion axis. The tibial markers were positioned in such a way that the knee and ankle joint centers and the ankle flexion/extension axis remain coplanar. Similarly, the thigh markers (LTHI and RTHI) were placed over the lower lateral 1/3 surface of the thigh, just below the swing of the hand, although the height is not critical. The thigh markers were used to calculate the knee flexion axis location and orientation. The knee markers (LKNE and RKNE) were placed on the lateral epicondyle of the knees. The pelvis markers (LASI and RASI) were placed directly over the left and right anterior superior iliac spine. These were positioned medially to the anterior superior iliac spines (ASIS) at slight bony prominences to get the marker to the correct position due to the curvature of the abdomen. These markers have defined the pelvic axes. After placing all sixteen reflective markers at the technically correct positions, gait cycle data have been captured and stored with the five AMEX cameras and the VICON system. The near-infrared (NIR) ray imping on the reflective markers have identified the movement of the lower limb when the ray reflected back to the camera. Since the ray was NIR, it was not disturbed by any possible obstacles coming on its way. Two force plates were used to capture the pertinent forces during the experiment. The subject was asked to walk at a normal speed on a level ground, which was about 0.89 m/s. The data captured from the subject were further analyzed with VICON NEXUS 1.8.5 system and Microsoft Excel to obtain the spatial, temporal, kinematics, and kinetics information. The experiment was conducted in the Motion Analysis Laboratory at the Faculty of Engineering, University of Malaya.

### 4.2. Gait and Motion Analyses Results and Discussions

**Figure 5:**
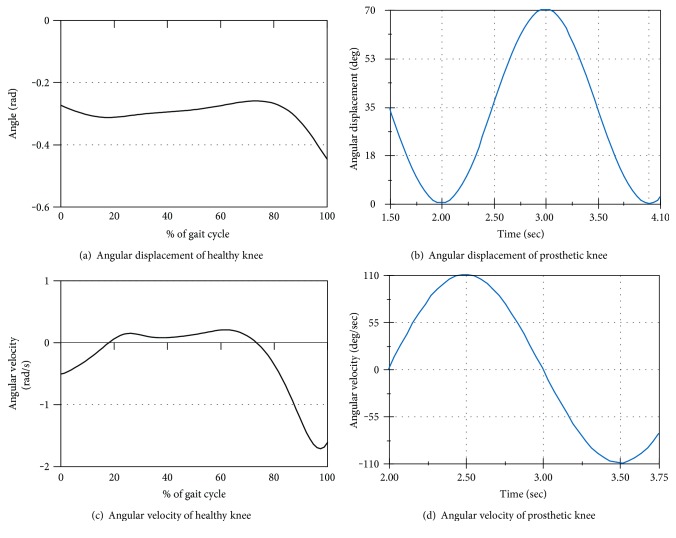
Angular displacement and angular velocity of the knee joint obtained from a healthy subject and from motion analysis of the prosthetic knee joint.

**Figure 6:**
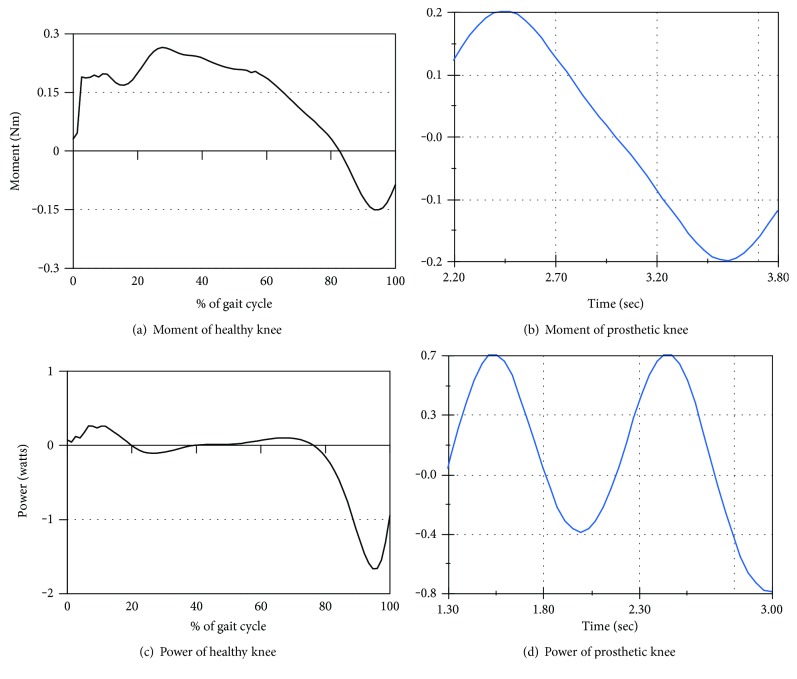
Joint moment and joint power of the knee joint obtained from a healthy subject and from motion analysis of the prosthetic knee.

A comparison between the experimental and predicted data has been performed, the results of which are shown in the following sections in Figures 5 and 6.

Figure 5(a) represents the changes in the angular displacement of the knee joint of a healthy biological lower limb during the gait cycle, whereas Figure 5(b) shows the angular displacement of the gears of the prosthetic knee joint for the same cycle. From these figures, the pattern of changing the knee angular displacement (Figure 5(a)) is quite similar to the shape of the graph of Figure 5(b). Though the magnitudes of angular displacements are not identical, the patterns of the graphs are observed to be alike. This is because the angular displacement varies based on walking speed, stepping length/stride length, and so forth; however, they still maintain a similar trend throughout the swing phase. Therefore, it can be deduced that the proposed gear-based knee joint is capable of reproducing the movement of a healthy biological knee joint effectively.

Figures 5(c) and 5(d) show the similarity or dissimilarity between the patterns of angular velocity graphs obtained from the data recorded from the healthy subject and the motion analysis result. The graph of Figure 5(c) is plotted based on the real data recorded from the healthy subject whereas the graph of Figure 5(d) is obtained from the motion analysis data. From Figures 4(c) and 5(d), the pattern of the angular velocity graph (Figure 5(c)) is similar to the pattern of the angular velocity profile of Figure 5(d). Though the exact values of both the graphs are not identical, their patterns have quite a similarity. Both the graphs are dynamic type; however, there are some phase differences, which can be attributed to the different rates of walking speed. Since the similar velocity was not maintained during simulation and subject's walking, there is some phase difference between the velocity graphs obtained from real data and from the motion analysis.

The joint moment and joint power graphs plotted with the real data (recorded from the healthy subject) have been compared with the reaction moment and joint power curves (obtained from the motion analysis of the knee joint), respectively. The performance of the prosthetic knee joint can be evaluated with the results of these analyses.

Figures 6(a) and 6(c) are plotted based on the real data taken from the healthy subject whereas Figures 6(b) and 6(d) are obtained from the motion analysis of the prosthetic knee joint. From Figures 6(a) and 6(b), the pattern of the joint moment curve is a dynamic-type signal; however, the nature of this graph has a great resemblance to the moment curve obtained from the kinetics analysis of the prosthetic knee joint. Though the magnitude of variation is not identical, the trends of the joint moment graphs are similar.

The fluctuation of joint power during the swing phase is shown in Figures 6(c) and 6(d). From the figures, the joint power at the different points of the swing phase varies both in positive and negative directions. The positive amplitude of the power graph represents the power generation by the knee joint whereas the negative amplitude shows the power absorption by the joint. From Figure 6(c), the pattern of the joint power curve produced from the real data is a dynamic-type signal. The pattern of the joint power curve (Figure 6(d)) obtained from the kinetics analysis of the knee joint is found to have a similar trend. Though the magnitude of the joint power is not identical, the pattern of the knee power curve of Figure 6(c) is similar to the pattern of the joint power curve of Figure 6(d). Due to the differences between the boundary conditions used for collecting data from the subject and simulation, there is some phase difference in the graphs. However, they are found to maintain a similar trend throughout the gait cycle.

## 5. Finite-Element Analysis of Knee Joint Components

Finite-element analysis (FEA) is carried out to optimize and validate each design step. The quality, performance, and safety of the product are also ensured based on the FEA results. Displacement, strain, and stress of the components under internal and external loads are calculated using the displacement formula of the finite-element method in the SOLIDWORKS platform. The FE analysis of knee joint components is carried out by linear stress analysis. The FEA is performed to confirm the geometry remains within the linear elastic range (meaning that the component can regain its original shape when the load is removed). The linear stress analysis of the components is carried out until the displacements, and rotations become small relative to the geometry. Factor of safety (FoS) is typically a design goal for this kind of analysis.

First of all, a model has to be created to do an FE analysis. Numbers of researchers have developed an FE model for prosthetic joints like the knee joint, ankle joint, hip joint, and temporomandibular joint and also for implants like an entire knee replacement [4]. To create a finite-element model, boundary conditions have to be set in the first place; the mesh formation is to be done next, and finally the simulation has to run to get the results. The detail of the knee joint modeling is given below.

### 5.1. Model Setup

The finite-element model of the knee joint was developed to check the functionality of the joint designed for the prosthesis. To set up a model, connections between the components, component contacts, fixtures, and external load are the primary factors to be defined accurately. The whole gear assembly is defined as a global contact, where the contact between the gears (gear 1 and gear 2), gears and stoppers, and gears and bearing plates is assigned as a bonded contact and the contact between the stoppers (stopper 1 and stopper 2) and between the bushing pins and bearings are defined as no penetration contact. The bearing plates are set up as fixed geometry where the faces between the gears and stoppers with the bearing plates are defined as the roller slider joint. The faces between the bushing pins and the bearing are set up as fixed hinge joints. Normal loads are applied from the top and bottom of gear 1 and gear 2, respectively. Two opposite directional torques are applied on the face of the two gears.

All components are considered solid in body. The aluminum alloy 1060-H16 is chosen for all components except for the ball bearing, sleeve bearing, and bushing pin, for which alloy steel (SS), copper alloy—brass, and alloy steel AISI 4140 are, respectively, selected. The properties of these materials are given in Table 3.

The key features of FE modeling of the knee joint components are tabulated in Table 4.

Boundary conditions used in the FEA of the prosthetic knee joint are shown in Table 5.

**Figure 7:**
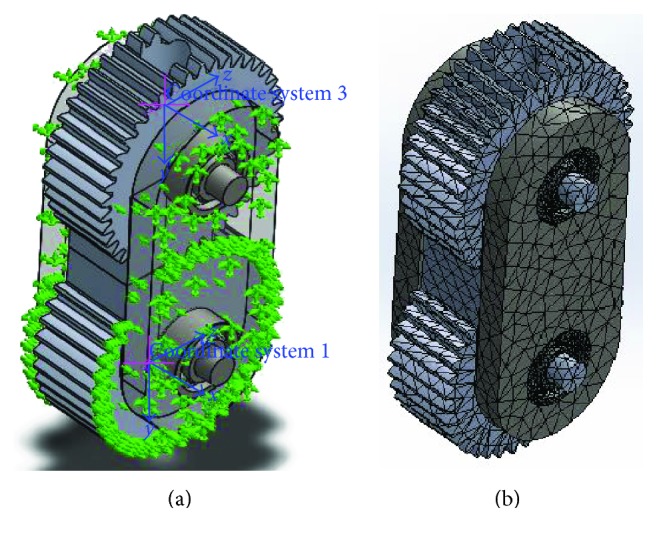
(a) Model of gear-based knee joint. (b) Solid mesh of the model.

Figure 7(a) illustrates all the boundary conditions and the external loads applied to the different components, and Figure 7(b) shows the meshing of the knee components.

### 5.2. Finite-Element Analysis Results and Discussions

In the finite-element analysis, the stress, strain, and displacement of the knee components are evaluated to check the viability of the design. In most of the engineering design, the maximum von Mises stress is calculated as a mean of design safety. The maximum von Mises stress is derived from the von Mises-Hencky theory, which is also recognized as the shear-energy theory or the maximum distortion energy theory. According to the theory, yielding of a ductile material starts when the von Mises stress and the stress limit become equal. The yield strength is usually taken as the limit of stress.

(17) $$\begin{array}{c} \sigma_{vonMises}\geq \sigma_{limit}. \end{array}$$

The yield strength of material largely depends on temperature. Therefore, the value of yield strength should take the temperature into consideration. The following equation can help to calculate the factor of safety at a certain location:

(18) $$\begin{array}{c} Factor\;of\;safety\;\left(FoS\right)=\frac{\sigma_{limit}}{\sigma_{vonMises}} \end{array}$$

**Figure 8:**
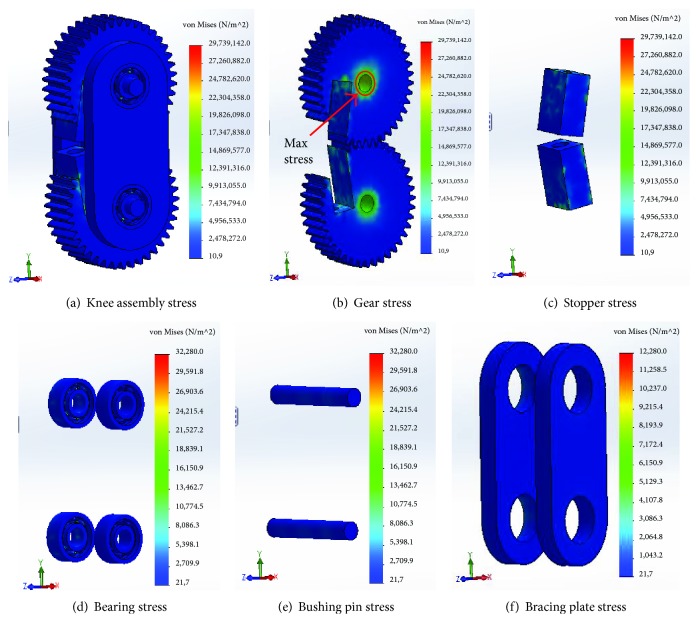
von Mises stress of the knee joint components.

The von Mises stresses of different knee components found from the finite-element analysis are shown in Figure 8.

The condition of plastic stability is the primary focus of the von Mises stress analysis. The results of the von Mises stress analysis show that the maximum stress of 29.74 MPa is obtained in gear (Figure 8), which remains below the maximum yield strength of material of 105 MPa (according to material properties of the 1060-H12 aluminum alloy). The factor of safety was 105.00/29.74 = 3.5, which is good enough to ensure the design safety. Therefore, no plastic deformation took place in the knee joint components, and the condition of plastic stability was complied.

The equivalent strain or the von Mises equivalent strain is a scalar quantity, which is another important factor for the design and is often used to describe the state of strain in solid components. The equivalent strain is commonly defined on plasticity, which is expressed as follows:

(19) $$\begin{array}{c} \varepsilon_{eq}=\sqrt{\frac{2}{3}\varepsilon^{dev}:\varepsilon^{dev}}=\sqrt{\frac{2}{3}\varepsilon_{ij}^{dev}\varepsilon_{ij}^{dev}},\varepsilon^{dev}=\varepsilon -\frac{1}{3}\mathrm{tr}\left(\varepsilon \right)1. \end{array}$$

This quantity is work conjugate to the equivalent stress defined as

(20) $$\begin{array}{c} \sigma_{eq}=\sqrt{\frac{3}{2}\sigma^{dev}:\sigma^{dev}}. \end{array}$$

**Figure 9:**
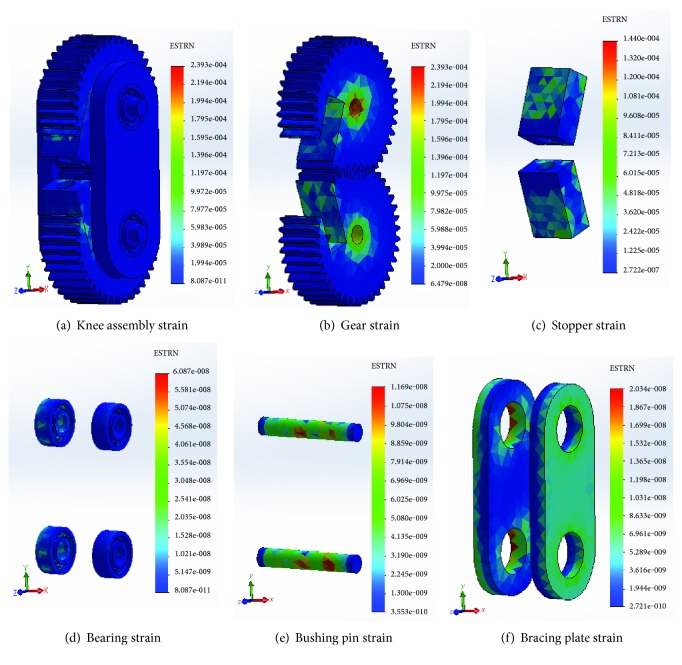
Strain analysis of the knee joint components.

The equivalent strains of different components of knee joints are shown in Figure 9.

From the equivalent strain calculations, it is obvious that the maximum strain of 2.393e−004 occurred in the gear (Figure 9), which is insignificant to appear as a distortion on the shape of the component and therefore can be neglected. Hence the components of the knee joint have met the condition of rigidity.

The displacement analysis allows one to assess the displacement and reaction force results for static, nonlinear, dynamic, drop test studies, or mode shapes for buckling and frequency studies.

**Figure 10:**
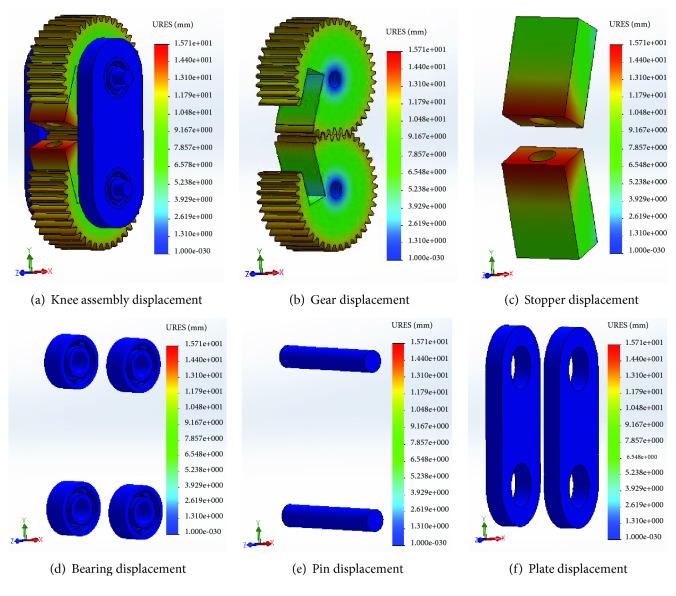
Displacement analysis of the knee joint components.

The results of static displacement studies are shown in Figure 10.

From Figure 10, the maximum static displacement of 7.97549 mm is found in the stopper during the analysis, which is also insignificant in magnitude and safe enough for the design. Therefore, the design has met the condition of stiffness as well.

From the finite-element analysis, the maximum von Mises stress, equivalent strain, and displacement of the components which occurred during the gait cycle of the prosthetic knee joint are found to remain quite below the material yield strength, permissible strain, and displacement limits, respectively. Therefore, the proposed design is safe for the particular application, and no unexpected failure will take place.

## 6. Conclusions

The gear-based knee joint designed for a transfemoral amputee can recreate gait cycle movement of a healthy biological limb and can be used compatibly with adequate safety. According to the motion study results, the prosthetic knee joint is capable of following the residual limb movement and thus enables the prosthesis to imitate the biomechanics of the missing limb without any difficulty. The maximum von Mises stress, equivalent strain, and static displacement experienced by the knee joint components are 29.74 MPa, 2.393e−004, and 7.97549 mm, respectively, which are much below the allowable limits of the materials used for making the components. The finite-element analysis result shows that the design is safe enough to be used by that particular subject.

## Figures and Tables

**Table 1:** Anthropometrical variables and subject characteristics.

Variable	Mean value
Subject height (cm)	166.5
Mass (kg)	69
Thigh/femur length (cm)	51.5
Shank/tibia length (cm)	41.0
Ankle-heel distance (cm)	8.6

**Table 2:** Boundary conditions of the knee joint simulation.

Constraints	Values
Motor rotation	Oscillating
Frequency	0.5 Hz
Angle of rotation by each gear	0°~7.5°, and 0°~35°

**Table 3:** Properties of the materials.

Material	Property				
	Elastic modulus (N/m^2^)	Poisson's ratio	Shear modulus (N/m^2^)	Tensile strength (N/m^2^)	Yield strength (N/m^2^)
Aluminum alloy 1060-H16	6.9e+010	0.33	2.6e+010	110000000	105000000
Alloy steel (SS)	2.1e+011	0.28	7.9e+010	723825617	620421998
Copper alloy—brass	1e+011	0.33	3.7e+010	478413000	239689000
Alloy steel AISI 4140	2.1e+011	0.28	7.9e+010	723825617	620421998

**Table 4:** Key features of finite-element modeling of knee joint components.

Various features of the FE model	
Software used	SOLIDWORKS
Solver type	FFEPlus
Mesh type	Solid mesh
Mesher used	Curvature-based mesh
Jacobian points	16 points
Element type	Triangular (2D)
Maximum element size	6.61898 mm
Minimum element size	1.3238 mm
Mesh quality	High
Remesh failed parts with incompatible mesh	On
Total nodes	95294
Total elements	53492
Maximum aspect ratio	26.613

**Table 5:** Boundary conditions of the knee joint FE analysis.

Constraints	Values	
	At 15° of rotation	At 70° of rotation
Load applied	691.6 N to 685.7 N	691.6 N to 566.5 N
Moment	Gear 1: 0 Nm to 46.5 Nm; gear 2: 0 Nm to 37.0 Nm	Gear 1: 0 Nm to 204.3 Nm; gear 2: 0 Nm to 162.6 Nm
